# Association Between Steno-Occlusive Middle Cerebral Artery and Basal Ganglia Perivascular Spaces

**DOI:** 10.3389/fneur.2020.00293

**Published:** 2020-04-23

**Authors:** Houwei Du, Chao Chen, Chengbin Ye, Feifei Lin, Jin Wei, Pincang Xia, Ronghua Chen, Sangru Wu, Qilin Yuan, Hongbin Chen, Yingchun Xiao, Nan Liu

**Affiliations:** ^1^Department of Neurology, Stroke Research Center, Fujian Medical University Union Hospital, Fuzhou, China; ^2^Department of Neurology, Fuzhou Second Hospital Affiliated to Xiamen University, Fuzhou, China; ^3^Department of Radiology, The People's Hospital of Fujian Traditional Chinese Medicine University, Fuzhou, China; ^4^Department of Radiology, Fujian Medical University Union Hospital, Fuzhou, China; ^5^School of Health Sciences, College of Health and Medicine, University of Tasmania, Newham, VIC, Australia; ^6^Department of STDs and HIV/AIDS Control and Prevention, Fujian Province Center for Disease Control and Prevention, Fuzhou, China; ^7^Department of Rehabilitation, Fujian Medical University Union Hospital, Fuzhou, China

**Keywords:** enlarged perivascular spaces, basal ganglia, cerebral small vessel disease, steno-occlusive middle cerebral artery, magnetic resonance image, computed tomography angiography, atherosclerosis

## Abstract

**Objectives:** Enlarged perivascular spaces in the basal ganglia (BG-EPVS) share common vascular risk factors with atherosclerosis. However, little is known about the relationship between steno-occlusive middle cerebral artery (MCA) and BG-EPVS. In this cross-sectional study, we aimed to test the hypothesis that severe MCA stenosis or occlusion is associated with increased MRI-visible BG-EPVS.

**Methods:** We retrospectively reviewed 112 patients with a steno-occlusive MCA from Fujian Medical University Union Hospital between January 2014 and December 2018. We rated BG-EPVS, white matter hyperintensities (WMH), and lacunes as markers of cerebral small vessel disease (CSVD) on magnetic resonance image (MRI). The severity of steno-occlusive MCA was assessed by computed tomography angiography (CTA) and was classified into moderate (50–69%), severe (70–99%), and occlusion (100%). We evaluated the association of steno-occlusive MCA for >10 BG-EPVS using logistic regression model adjusted for age, gender, hypertension, MR-visible WMH, and lacunes. We also compared the number of BG-EPVS between the affected side and unaffected side in patients with only unilateral steno-occlusive MCA.

**Results:** In multivariable logistic regression analysis, age (OR = 1.07, 95%CI: 1.03–1.13, *p* = 0.003), hypertension (OR = 2.77, 95%CI: 1.02–7.51, *p* = 0.046), severe MCA stenosis (OR = 3.65, 95%CI: 1.12–11.87, *p* = 0.032), or occlusion (OR = 3.67, 95%CI: 1.20–11.27, *p* = 0.023) were significantly associated with >10 BG-EPVS. The number of BG-EPVS in the affected side was higher than the unaffected side in patients with severe MCA stenosis (12 [9−14] vs. 8 [6−11], *p* = 0.001) or occlusion (11 [7−14] vs. 8 [5−11], *p* = 0.028).

**Conclusions:** BG-EPVS were more prevalent in patients with severe MCA atherosclerosis. Our findings suggest a biological link between severe steno-occlusive MCA and increased BG-EPVS. These results need confirmation in prospective studies.

## Introduction

Perivascular spaces (PVS) refer to tiny fluid-filled cavities surrounding the cerebral small perforating blood vessels that penetrate the brain parenchyma (1). PVS are normally invisible on conventional magnetic resonance image (MRI) and become visible on neuroimaging when PVS are presumably prominent or dilated (2). Basal ganglia (BG) is a common site for enlarged perivascular spaces (EPVS) formation where the largest cerebrospinal fluid (CSF) influxes occur along large ventral perforating arteries (3, 4). Till date, the underlying pathogenetic mechanisms involved in enlarged perivascular spaces in the basal ganglia (BG-EPVS) remain unresolved. Previous studies have demonstrated that BG-EPVS are involved in physiological mechanisms of the drainage of interstitial fluid to the systemic circulation (1, 5). Therefore, conditions elevating intraluminal pressure within these perforating arteries that compromise this drainage system may cause BG-EPVS (6).

Recently, the importance of understanding the pathophysiology of BG-EPVS in cerebrovascular disease is highlighted by their association with cerebral small vessel disease (CSVD), large artery atherosclerosis, and risk of stroke (7–9). Middle cerebral artery (MCA) is among the common locations of intracranial artery atherosclerosis, and MCA atherosclerosis is increasingly thought to be a significant precipitant of cerebral ischemic stroke, particularly in the Asian population (10–12). Impaired arterial stiffness (13) and increased pulsatility index (14) were found in severe MCA stenosis. Moreover, previous studies showed increased cerebrovascular pulsatility and arterial stiffness were related to increased BG-EPVS (15–17). Based on these findings, it is reasonable to imagine that BG-EPVS might be more prevalent in patients with severe MCA atherosclerosis. MCA gives rise to the deep perforating branches (also known as lenticulostriate arteries) that supply the basal ganglia where BG-EPVS appear along the lenticulostriate arteries through the anterior perforated substance on MRI (3, 4, 18). Therefore, investigating the relationship between BG-EPVS and MCA atherosclerosis might help understand pathogenetic mechanisms and clinical significance of BG-EPVS. However, to our knowledge, this association remains poorly understood. In the present study, we aimed to test the hypothesis that steno-occlusive MCA is associated with the presence of MR-visible BG-EPVS in a Chinese population.

## Materials and Methods

### Population

We retrospectively reviewed demographic characteristics and MRIs of patients with MCA stenosis disease between January 2014 and December 2018 who were treated in Fujian Medical University Union Hospital if they met the following criteria: (a) age ≥ 18 years; (b) moderate (50–69%) to severe (70–99%) atherosclerotic MCA stenosis or occlusion (100%) defined by contrast-enhanced cerebral vessel computed tomography angiography (CTA); (c) <50% stenosis in internal carotid artery; (d) no history of intravascular intervention or surgical treatment for steno-occlusive MCA disease. The exclusion criteria were: (a) MCA stenosis or occlusion associated with Moyamoya disease, arteriovenous malformations, carotid dissection, primary vasculitis of the central nervous system or other etiologies than atherosclerosis; (b) patients with extensive stroke in the basal ganglia or other diseases that may affect accurate assessment of BG-EPVS on MRI, including severe hydrocephalus, subarachnoid hemorrhage, intracranial infection, multiple sclerosis, history of brain surgery; (c) low-quality of MRI images leading to failing to assess BG-EPVS, white matter hyperintensities (WMH), or lacunes. The study protocol was approved by Fujian Medical University Union Hospital ethics committee, and patient informed consent was waived due to the study design.

### Clinical Data

The demographic and radiological characteristics of the participants, including their vascular risk factors, MRI and CTA features were collected from a database of Fujian Medical University Union Hospital. The known risk factors included hypertension (defined as receiving medications for hypertension or blood pressure >140/90 mmHg on repeated measurements), diabetes mellitus (defined as receiving medications for diabetes mellitus, fasting blood glucose ≥7.0 mmol/L or HbA1c ≥6.5%, or a casual plasma glucose >11.1 mmol/L), ischemic heart disease, dyslipidemia (defined as an overnight fasting cholesterol level ≥6.2 mmol/L, ≥2.3 mmol/L triglycerides, low-density lipoprotein (LDL) cholesterol ≥4.1 mmol/L, or high-density lipoprotein (HDL) cholesterol ≤1.0 mmol/L).

### MR Imaging

MRI was performed on a 1.5T MRI system (Symphony Vision, Siemens Health Care, Germany) or a 3.0T MRI (Discovery MR750, GE Healthcare, USA) by using a standardized protocol. Slice thickness was 5 mm with 1.5 mm gap between slices. Parameters for 1.5T scanner, T1WI sequence: repetition time (TR), 1,990 ms; echo time (TE), 8.7 ms, field of view (FOV), 230 × 217 mm^2^; T2WI sequence: TR, 4,700 ms; TE, 109 ms; FOV, 230 × 217 mm^2^; fluid-attenuated inversion recovery (FLAIR) sequence: TR, 9,000 ms; TE, 95 ms; FOV, 230 × 217 mm^2^; diffusion-weighted imaging (DWI) sequence: TR, 3,570 ms TE, 67 ms, FOV, 235 × 235 mm^2^; Parameters for 3.0T scanner, T1WI sequence: TR, 2,925.9 ms; TE, 24 ms, FOV, 240 × 192 mm^2^; T2WI sequence: TR, 4,929 ms; TE, 105 ms; FOV, 240 × 240 mm^2^; FLAIR sequence: TR, 8,500 ms; TE, 140 ms; FOV, 240 × 224 mm^2^; DWI sequence: TR, 3,000ms; TE, minimum; FOV 240 × 240 mm^2^. MRI markers of CSVD, including BG-EPVS, WMH, and lacunes were assessed blinded to clinical information by a trained neuroradiologist (JW), according to previous literature (19, 20). BG-EPVS were defined as round or linear hyperintense lesions with <3 mm in size on T2-weighted images in the basal ganglia slice above the level of the anterior perforated substance or substantia innominata (https://www.ed.ac.uk/files/imports/fileManager/epvs-rating-scale-user-guide.pdf) (21). According to previously published method, BG-EPVS were classified based on the following scale: grade 0 = no EPVS; grade 1 = 1–10 EPVS; grade 2 = 11–20 EPVS; grade 3 = 21–40 EPVS; and grade 4 = >40 EPVS, and were defined as abnormal if grade 2–4 (number >10) were present (20, 22, 23). The extent of WMH was determined on the FLAIR images of periventricular white matter or deep white matter according to Fazekas' scoring system (24). A Fazekas score ≥3 in periventricular white matter and/or ≥2 in deep white matter were defined as moderate to severe WMH (25). Lacunes were defined as one or more rounded or ovoid cavitary lesions 3–20 mm in diameter, with high-signal intensity on T2 FLAIR and T2-weighted images and low signal intensity on T1-weighted images (20).

### CTA Assessment

CTA was performed on all participants, using a 64-slice CT scanner (Discovery CT 750 HD, GE Health-care, Milwaukee, WI) with tube voltage at 120 kVp, automatic tube current modulation and slice thickness of 0.625 mm. The contrast enhancement was achieved by intravenous injection of 40–60 mL contrast agent (Iohexol, 320 mg I/mL, Hengrui Medicine, Lianyungang, China) at 4–5 mL/s injection rate. CTA images were further processed using thin-slice maximum intensity projection (TS-MIP), multiplanar reconstruction (MPR), and volume rendering (VR) functions with the software packages of Volume Viewer. TS-MIP and MPR were used to evaluate stenosis and visualize the lumen and wall of the vessel (26). All CTA images were assessed blinded to clinical information by a certified radiologist (CY). The severity of MCA stenosis was calculated by the following equation according to the Warfarin Aspirin Symptomatic Intracranial Disease (WASID) criteria (27): degree of stenosis (%) = (1 – [*D*_stenosis_/*D*_normal_]) × 100%, where *D*_stenosis_ was the luminal diameter of the narrowest part of MCA and *D*_normal_ was the diameter of the proximal normal artery. *D*_normal_ was determined by the following criteria: the diameter of the proximal part of the MCA at its widest, non-tortuous, normal segment was chosen (first choice). If the proximal MCA was diseased, the diameter of the distal portion of the MCA at its widest, parallel, non-tortuous normal segment was substituted (second choice). If the entire MCA was diseased, the most distal, parallel, non-tortuous normal segment of the feeding artery which referred to the supraclinoid carotid artery was measured (third choice) (26). Due to the differences in anteroposterior projection of the CTA images, the variability of the vasculature size and slight differences of image magnification, the contralateral circulation was not recommended to be determined as the “normal” reference artery (28). We defined the side with steno-occlusive MCA as the affected side and the side without steno-occlusive MCA as the unaffected side.

### Statistics

Categorical variables are summarized as absolute numbers with percentages, and continuous variables as means with standard deviations (SD) if normally distributed or median with inter-quartile range (IQR) if not normally distributed. Considering that BG-EPVS were not normally distributed, we dichotomized BG-EPVS into 0–10 (EPVS grade 0–1) and >10 (EPVS grade 2–4), which mirrors mild versus moderate to severe EPVS to permit binary logistic regression (7). Chi-square test or Fisher's exact test, and Student *t*-test where appropriate are used to compare the difference in clinical and neuroimaging characteristics between 0–10 and >10 BG-EPVS. Candidate variables, selected based on medical knowledge and previous reports, included age, gender, conventional vascular risk factors, and MRI markers of WMH and lacunes. We calculated the odds ratio (OR) with 95% confidence interval (CI) of steno-occlusive MCA for >10 BG-EPVS using univariate logistic analysis. We then included variables of *P* < 0.2 in the univariate analysis as confounders into the multivariable logistic regression analysis. Wilcoxon rank-sum test was used to compare the numbers BG-EPVS between the affected and unaffected sides in the participants with only unilateral steno-occlusive MCA. *P* < 0.05 was considered statistically significant. All statistics were done using SPSS 25.0 (SPSS Inc., Chicago, IL, USA).

## Results

### Clinical Demographics, MRI, and CTA Characteristics

**Figure 1:**
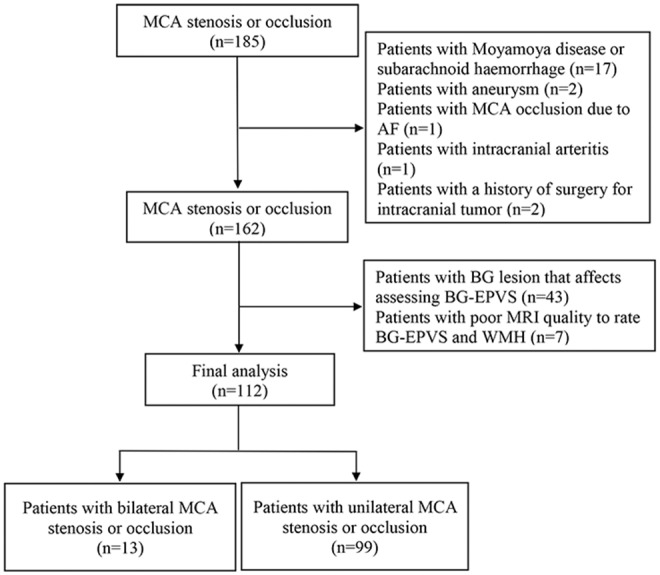
Flow chart of patient's selection. MCA, middle cerebral artery; AF, Atrial fibrillation; BG, basal ganglia; EPVS, enlarged perivascular spaces.

We included 185 consecutive patients with steno-occlusive MCA diagnosed by CTA, who also underwent conventional brain MRI between January 2014 and December 2018. After excluding 21 patients with non-atherosclerotic MCA stenosis or occlusion, 2 patients with a history of brain tumor surgery, 43 patients with basal ganglia lesion that affects the assessment of BG-EPVS, and 7 patients with poor MRI quality to rate BG-EPVS and WMH, 112 eligible participants (91 with stroke defined by clinical evaluation and MRI-DWI positive findings; 21 were stroke-free) were included in the final analysis (Figure 1). A total of 99 patients were diagnosed with only unilateral MCA stenosis or occlusion. Clinical demographics, MRI, and CTA characteristics of the participants are shown in Table 1. The mean age of the participants was 63.0 (11.0) years, and 77 (68.8%) of them were male. Hypertension was present in 76 (67.9%) patients, diabetic mellitus in 40 (35.7%), dyslipidemia in 63 (56.3%), current smoking in 23 (20.5%), and ischemic heart disease in 12 (10.7%) patients. Forty-one (36.6%) patients had moderate MCA stenosis, 32 (28.6%) patients had severe MCA stenosis, and 39 (34.8%) patients had MCA occlusion. Looking at MRI markers of CSVD, 44 (39.3%) patients had 0–10 BG-EPVS, 57 (50.9%) patients had 11–20 BG-EPVS, 11 (9.8%) patients had >20 BG-EPVS. We found moderate-to-severe WMH in 27 (24.1%) patients, lacunes in 54 (48.2%) patients. The intra-rater agreement was assessed on a random sample of 50 individuals with 1-month interval between the first and second image evaluation. Intra-rater reliability for CSVD markers and MCA stenosis were excellent: BG-EPVS (κ = 0.92, 95%CI: 0.86–0.98); lacunes (κ = 0.92, 95%CI: 0.88–1.03); WMH (κ = 0.91, 95%CI: 0.78–1.03); and MCA stenosis (κ = 0.93, 95%CI: 0.86–1.01).

**Table 1:** Clinical, demographic, and radiological characteristics of the participants.

**Variables**	
Age (year), mean (SD)	63.0 ± 11.1
Male, *n* (%)	77 (68.8)
**Smoker**, ***n*** **(%)**	
None	71 (63.4)
Ex	18 (16.1)
Current	23 (20.5)
IHD, *n* (%)	12 (10.7)
Hypertension, *n* (%)	76 (67.9)
DM, *n* (%)	40 (35.7)
Dyslipidemia, *n* (%)	63 (56.3)
**MCA stenosis (%)**	
50–69, *n* (%)	41 (36.6)
70–99, *n* (%)	32 (28.6)
100, *n* (%)	39 (34.8)
Magnet strength, 1.5T, *n* (%)	83 (74.1)
Lacunes, *n* (%)	54 (48.2)
Moderate to severe WHM, *n* (%)	27 (24.1)
**BG-EPVS**, ***n*** **(%)**	
0–10	44 (39.3)
11–20	57 (50.9)
21–40	10 (8.9)
>40	1 (0.9)

*SD, standard deviations; MCA, middle cerebral artery; IHD, ischemic heart disease; DM, diabetes mellitus; T, Tesla; WMH, white matter hyperintensities; BG-EPVS, enlarged perivascular spaces in the basal ganglia*.

### Difference Between 0–10 and >10 BG-EPVS

Comparison of characteristics between patients with 0–10 and >10 BG-EPVS is shown in Table 2. Participants with >10 BG-EPVS were older (66.1 ± 10.0 vs. 58.0 ± 11.1), more likely to be male (73.5 vs. 61.4%), more likely to have hypertension (77.9 vs. 52.3%), severe MCA stenosis (33.8 vs. 20.5%), occlusive MCA (36.8 vs. 31.8%), lacunes (58.8 vs. 31.8%), and moderate-to-severe WMH (30.9 vs. 13.6%).

**Table 2:** Characteristics between 0–10 and >10 BG-EPVS groups.

	**0–10 BG-EVPS (*n* = 44)**	**>10 BG-EPVS (*n* = 68)**	***p***
Age, year (SD)	58.0 ± 11.1	66.1 ± 10.0	<0.001
Male, *n* (%)	27 (61.4)	50 (73.5)	0.175
Smoker, *n* (%)			0.874
None, *n* (%)	29 (65.9)	42 (61.8)	
Ex, *n* (%)	7 (15.9)	11 (16.2)	
Current, *n* (%)	8 (18.2)	15 (22.1)	
IHD, *n* (%)	4 (9.1)	8 (11.8)	0.655
Hypertension, *n* (%)	23 (52.3)	53 (77.9)	0.005
DM, *n* (%)	14 (31.8)	26 (38.2)	0.489
Dyslipidemia	24 (54.5)	39 (57.4)	0.770
MCA stenosis (%)			0.116
50–69, *n* (%)	21 (47.7)	20 (29.4)	
70–99, *n* (%)	9 (20.5)	23 (33.8)	
100, *n* (%)	14 (31.8)	25 (36.8)	
Magnet strength, 1.5T, *n* (%)	34 (77.3)	49 (72.1)	0.538
Lacunes, *n* (%)	14 (31.8)	40 (58.8)	0.005
Moderate-to-severe WMH, *n* (%)	6 (13.6)	21 (30.9)	0.037

*SD, standard deviations; MCA, middle cerebral artery; BG-EPVS, enlarged perivascular spaces in the basal ganglia; IHD, ischemic heart disease; DM, diabetes mellitus; T, Tesla; WMH, white matter hyperintensities*.

### Logistic Regression Analysis

In univariable logistic regression analysis, age (OR = 1.08, 95%CI: 1.03–1.12, *p* < 0.001), hypertension (OR = 3.23, 95%CI: 1.42–7.35, *p* = 0.005), lacunes (OR = 3.06, 95%CI: 1.38–6.79, *p* = 0.006) and moderate-to-severe WMH (OR = 2.83, 95%CI: 1.04–7.72, *p* = 0.042) were significantly associated with >10 BG-EPVS. In multivariable logistic regression analysis, age (OR = 1.07, 95%CI: 1.03–1.13, *p* = 0.003), hypertension (OR = 2.77, 95%CI: 1.02–7.51, *p* = 0.046), severe MCA stenosis (OR = 3.65, 95%CI: 1.12–11.87, *p* = 0.032), or occlusion (OR = 3.67, 95%CI: 1.20–11.27, *p* = 0.023) were significantly associated with >10 BG-EPVS. Lacunes (OR = 2.62, 95%CI: 0.97–7.07, *p* = 0.059) were marginally associated with >10 BG-EPVS (Table 3). The association between severe MCA stenosis (adjusted OR = 3.73, 95%CI: 1.14–12.21, *p* = 0.030) or occlusion (adjusted OR = 3.42, 95%CI: 1.09–10.72, *p* = 0.035) remained consistent and of similar effect size with additional adjustment for different MRI scanners. As a sensitivity analysis in those with only unilateral MCA stenosis or occlusion (*n* = 99), severe MCA stenosis (adjusted OR = 3.79, 95%CI: 1.09–13.16, *p* = 0.036) or occlusion (adjusted OR = 4.86, 95%CI: 1.50–15.77, *p* = 0.008) remained associated with >10 BG-EPVS. Additional adjustment for different sides had a negligible effect on the association between severe MCA stenosis (adjusted OR = 4.77, 95%CI: 1.25–18.25, *p* = 0.022) or occlusion (adjusted OR = 4.62, 95%CI: 1.38–15.48, *p* = 0.013) and >10 BG-EPVS.

**Table 3:** Association of severe MCA stenosis/occlusion and >10 BG-EPVS.

	**Unadjusted**		**Adjusted**	
	**OR (95%CI)**	***P***	**OR (95%CI)**	***P***
Age	1.08 (1.03, 1.12)	<0.001	1.07 (1.03, 1.13)	0.003
Male	1.75 (0.78, 3.94)	0.177	2.20 (0.84, 5.77)	0.110
Hypertension	3.23 (1.42, 7.35)	0.005	2.77 (1.02, 7.51)	0.046
MCA stenosis (%)		0.121		0.036
50–69	Ref	Ref	Ref	Ref
70–99	2.68 (1.00, 7.18)	0.049	3.65 (1.12, 11.87)	0.032
100	1.88 (0.77, 4.59)	0.169	3.67 (1.20, 11.27)	0.023
Lacunes	3.06 (1.38, 6.79)	0.006	2.62 (0.97, 7.07)	0.059
Moderate-to-severe WMH	2.83 (1.04, 7.72)	0.042	1.40 (0.42, 4.68)	0.586

*MCA, middle cerebral artery; BG-EPVS, enlarged perivascular spaces in the basal ganglia; IHD, ischemic heart disease; DM, diabetes mellitus; WMH, white matter hyperintensities*.

### Comparison of Numbers of BG-EPVS

Comparison of number of BG-EPVS between the affected and the unaffected side in those with only unilateral MCA stenosis or occlusion are shown in Table 4. The number of BG-EPVS was higher in the affected side than the unaffected side in patients with severe MCA stenosis (12 [IQR 9–14] vs. 8 (6–11), *p* = 0.001) and occlusion (11 [IQR 7–14] vs. 8 (5–11), *p* = 0.028). We found no significant difference in the number of MR-visible BG-EPVS between affected and unaffected side in patients with moderate MCA stenosis (8 [IQR 5–11] vs. 8 (5–11), *p* = 0.173).

**Table 4:** Comparison of numbers of BG-EPVS in the affected and unaffected side.

**Unilateral MCA stenosis/occlusion**	**BG-EPVS (M [IQR])**		
	**Affected**	**Unaffected**	***P***
Moderate (*n* = 35)	8 (5–11)	8 (5–11)	0.173
Severe (*n* = 26)	12 (9–14)	8 (6–11)	0.001
Occlusion (*n* = 38)	11 (7–14)	8 (5–11)	0.028

*BG-EPVS, enlarged perivascular spaces in the basal ganglia; M, median; IQR, inter-quartile range*.

## Discussion

In this study of 112 participants with moderate to severe MCA stenosis or occlusion, we demonstrated an association between severe steno-occlusive MCA and >10 BG-EPVS, which was beyond the effect of age, gender, hypertension, and MRI markers such as WMH and lacunes. Moreover, the number of BG-EPVS was higher in the affected side than the unaffected side of severe steno-occlusive MCA.

To the best of our knowledge, our present study is the first to focus on the association between MCA atherosclerosis and BG-EPVS in a Chinese population. Although we cannot directly compare our data with those from others, previous studies addressing the relationship between BG-EPVS and carotid atherosclerosis (8, 29, 30) may provide important evidence to support our findings. It seems unlikely that the development of BG-EPVS causes MCA atherosclerosis, but the underlying pathophysiologic mechanisms remain unclear. One possible explanation for the association of BG-EVPS and atherosclerosis might be cerebral hypoperfusion (8, 29). Our findings are supported by a previous study that showed hemodynamically compromised hemispheres tend to demonstrate a higher number of EPVS (31). However, the sample size (*n* = 28) of the previous study (31) is smaller than our study and unusable for association analysis. Affected cerebral blood flow was observed in patients with severe steno-occlusive MCA (12, 26), and there is evidence that reduced cerebral blood flow is a factor in CSVD (32). Contrastly, cerebral blood flow was not significantly affected in patients with moderate MCA stenosis (26), which might account for the negative association between moderate MCA stenosis and BG-EPVS in our study. Moreover, previous experimental studies showed hypoperfusion secondary to internal carotid artery ligation (33) or MCA occlusion (34) caused impaired CSF influx. Based on these findings, we propose that hypoperfusion caused by severe-occlusive MCA could trigger hypoxia (12, 14, 35), impair the interstitial fluid drainage system (1, 3), and in turn facilitate the formation of BG-EPVS (1, 8). This hypothesis needs to be tested in longitudinal studies.

The correlation between BG-EPVS and MCA atherosclerosis may represent a shared association with atherosclerotic risk factors. (9, 11, 29). Possible contributors to the development of BG-EPVS may be arterial stiffness (17) and cerebrovascular pulsatility (6, 36), since both conditions are related to atherosclerosis (29). Based on the tsunami wave model, wave damage to the land increases with the narrowing of a river; Atherosclerotic vascular narrowing and vascular stiffness may accelerate the pulse waves (37). A previous study showed that the cerebral arterial stiffness measured by cerebral pulse wave velocity was positively correlated with the degree of stenosis in the segment between the common carotid artery and the ipsilateral MCA (13). Since fluid flow along the brain drainage pathway is driven by cerebral arterial pulsatility (1), we considered the increase BG-EPVS on the stenosis side of the MCA might be related to the increased pulsatile flow that is propagated distally along the large arterial bed. This hypothesis is supported by a subgroup analysis from the Oxford Vascular Study that showed a strong association between MCA pulsatility index and an increasing CSVD burden (OR = 4.26, 95%CI: 1.45–12.55, *p* = 0.009) (38).

In line with previous population-based MR imaging studies (9, 29), our findings also support an association between hypertension and increased BG-EPVS, suggesting that BG-EPVS might be considered a marker of hypertensive arteriopathy. A possible explanation for this association is that an increase in intraluminal pressure might facilitate greater extravasation of fluid through the small penetrating arteries into their surrounding spaces (6). Prospective studies with long-term follow-up are needed to test this hypothesis.

BG-EPVS in our sample increased with age after adjusting for other covariates, suggesting that BG-EPVS might be a manifestation of aging. Our findings were in line with previous studies (39, 40). However, the association of aging with high degree of BG-EPVS has not been reproduced in healthy adults in the Kashima Scan Study (41). This discrepancy could be explained by differences in selected participants. Future studies with larger sample size enabling analyses stratified on narrower age ranges could be of interest.

Limitations and strengths of our study: First, we cannot evaluate the causality due to the small sample size cross-sectional study. Notably, patients who were unable to undergo CTA and MRI detection were excluded, which brings selection bias. Second, we lacked the information about cerebral hemodynamic parameters or structural-functional data of MCA, which needs to be involved in further studies. Strengths of our study include careful assessment of MRI features of CSVD and the severity of MCA stenosis by two experienced radiologists blinded to the knowledge of the clinical demographics, which might minimize expectation bias.

Our present study showed a biological link between severe steno-occlusive MCA and increased BG-EPVS, raising the possibility that BG-EPVS could be considered a potential biomarker of severe steno-occlusive MCA. Prospective studies are needed to confirm the importance of BG-EPVS in relation to clinical outcomes of cerebrovascular disorders.

## Data Availability Statement

The datasets analyzed in this article are not publicly available. Requests to access the datasets should be directed to Prof. Nan Liu, xieheliunan1984@sina.com.

## Ethics Statement

The studies involving human participants were reviewed and approved by Fujian Medical University Union Hospital Ethics Committee. The ethics committee waived the requirement of written informed consent for participation.

## Author Contributions

HD and CC equally contributed to this work and HD, including data analysis and drafting of the manuscript. HD, CC, and PX contributed to the statistical analysis and manuscript revision. FL, RC, SW, QY, YX, and HC contributed to the acquisition of the data. CY and JW involved in image analysis and manuscript revision. HD and NL contributed to the study conception and design, analysis and interpretation of data, and manuscript revision.

## Conflict of Interest

The authors declare that the research was conducted in the absence of any commercial or financial relationships that could be construed as a potential conflict of interest.

## Abbreviations

BG: basal ganglia
EPVS: enlarged perivascular spaces
CSVD: cerebral small vessel disease
MCA: middle cerebral artery
WMH: white matter hyperintensities
MRI: magnetic resonance image
CTA: computed tomography angiography
OR: odds ratio
CI: confidence interval
LDL: low-density lipoprotein
HDL: high-density lipoprotein
FLAIR: fluid-attenuated inversion recovery
DWI: diffusion-weighted imaging
TR: time repetition
TE: time echo
SD: standard deviations
IQR: inter-quartile range.
